# Increased Physiological GDNF Levels Have No Effect on Dopamine Neuron Protection and Restoration in a Proteasome Inhibition Mouse Model of Parkinson’s Disease

**DOI:** 10.1523/ENEURO.0097-22.2023

**Published:** 2023-02-07

**Authors:** Soophie Olfat, Kärt Mätlik, Jaakko J. Kopra, Daniel R. Garton, Vilma H. Iivanainen, Dipabarna Bhattacharya, Johan Jakobsson, T. Petteri Piepponen, Jaan-Olle Andressoo

**Affiliations:** 1 Division of Neurogeriatrics, Department of Neurobiology, Care Sciences and Society (NVS), Karolinska Institutet, Stockholm 17177, Sweden; 2 Department of Pharmacology, Faculty of Medicine, Neuroscience Center & Helsinki Institute of Life Science, University of Helsinki, Helsinki 00290, Finland; 3 Division of Pharmacology and Pharmacotherapy, Faculty of Pharmacy, University of Helsinki, Helsinki 00014, Finland; 4 Laboratory of Molecular Neurogenetics, Department of Experimental Medical Science, Wallenberg Neuroscience Center and Lund Stem Cell Center, BMC A11, Lund University, Lund 221 84, Sweden

**Keywords:** 3′UTR, dopamine, GDNF, mouse model, Parkinson’s disease

## Abstract

Parkinson’s disease (PD) is a progressive neurodegenerative disease that comprises a range of motor and nonmotor symptoms. Glial cell line-derived neurotrophic factor (GDNF) promotes the survival of dopamine neurons *in vitro* and *in vivo*, and intracranial delivery of GDNF has been tested in six clinical trials for treating PD. However, clinical trials with ectopic GDNF have yielded variable results, which could in part result from abnormal expression site and levels caused by ectopic overexpression. Therefore, an important open question is whether an increase in endogenous GDNF expression could be potent in reversing PD progression. Here, we tested the therapeutic potential of endogenous GDNF using mice in which endogenous GDNF can be conditionally upregulated specifically in cells that express GDNF naturally (conditional GDNF hypermorphic mice; *Gdnf^cHyper^*). We analyzed the impact of endogenous GDNF upregulation in both neuroprotection and neurorestoration procedures, and for both motor and nonmotor symptoms in the proteasome inhibitor lactacystin (LC) model of PD. Our results showed that upregulation of endogenous GDNF in the adult striatum is not protective in LC-induced PD model in mice. Since age is the largest risk factor for PD, we also analyzed the effect of deletion of endogenous GDNF in aged *Gdnf* conditional knock-out mice. We found that GDNF deletion does not increase susceptibility to LC-induced damage. We conclude that endogenous GDNF does not impact the outcome in the LC-induced proteasome inhibition mouse model of Parkinson’s disease.

## Significance Statement

Glial cell line-derived neurotrophic factor GDNF is a strong enhancer of dopamine neuron function and survival. Ectopic delivery of GDNF has been tested in six clinical trials to treat Parkinson’s disease (PD) with promising but inconclusive results. Whether an increase in endogenous GDNF expression could be more beneficial in protecting or restoring damaged dopamine neurons than ectopic GDNF delivery has remained unknown. Here, we use GDNF conditional hypermorph and GDNF conditional knock-out mice to study the role of endogenous GDNF in a mouse model of Parkinson’s disease. Our findings demonstrate that neither an increase or loss of endogenous GDNF expression impacts dopamine neuron survival and recovery in proteasome inhibition model of Parkinson’s disease.

## Introduction

Parkinson’s disease (PD) is a progressive neurodegenerative disease that affects >10 million people worldwide (Meissner et al., 2011). The classical motor symptoms of PD mostly result from a gradual degeneration of substantia nigra pars compacta (SNpc) dopaminergic neurons and the consequent loss of dopamine fibers in the dorsal striatum (DeMaagd and Philip, 2015). In addition, PD patients also display a range of nonmotor symptoms that have a significant impact on their quality of life. Dopaminergic neuron dysfunction and death in PD is believed to progress because of failure in mitochondria and proteostasis, which manifests as reduced proteasomal function in the SNpc (Bentea et al., 2017; Park et al., 2018). Current treatments are mainly limited to symptomatic treatment of motor dysfunction. No treatment is available to halt or reverse the progression of PD.

Glial cell line-derived neurotrophic factor (GDNF) promotes dopamine synthesis and turnover in cultured dopaminergic neurons and induces tyrosine hydroxylase (TH)-positive fiber outgrowth *in vivo* (Lin et al., 1993; Hoffer et al., 1994). Therefore, GDNF delivery to reverse dopaminergic degeneration has been tested in preclinical and clinical trials. Most preclinical studies have been performed using 1-methyl-4-phenyl-1,2,3,6-tetrahydropyridine (MPTP) and 6-hydroxydopamine (6-OHDA) in PD animal models, where toxins enter dopamine neurons via dopamine transporter (DAT) and acutely kill specifically the dopamine neurons (Lotharius et al., 1999). Based on success of those studies, six clinical trials with ectopic delivery of GDNF have been performed, with variable outcomes (Georgievska et al., 2002, 2004; Manfredsson et al., 2009; Meissner et al., 2011; Barker et al., 2020). Among unresolved questions is the optimal source of GDNF (Barker et al., 2020) and how to achieve the regrowth of dopamine neuron axons across the striatum (Ibáñez and Andressoo, 2017; Bondarenko and Saarma, 2021). Since neurotrophic factors, including GDNF, act as chemoattractants, ectopic GDNF delivery results in the regrowth of dopamine neuron axons toward the GDNF application site rather than toward their physiological innervation targets (Georgievska et al., 2002, 2004; Rosenblad et al., 2003; Love et al., 2005; Nagahara et al., 2009). Each dopamine neuron normally forms wide arborizations in the striatum and innervates up to 5% of the whole striatum volume (Matsuda et al., 2009). Endogenous GDNF is expressed in striatal interneurons that are sparsely located across the whole striatum (Hidalgo-Figueroa et al., 2012; d’Anglemont de Tassigny et al., 2015). For optimal therapeutic outcome, it might therefore be beneficial to increase dopaminergic innervation striatum-wide through enhancing chemoattraction from naturally GDNF expressing cells. However, the therapeutic potential of endogenous GDNF upregulation is unknown because of the lack of methods allowing to specifically upregulate endogenous GDNF in the adult striatum.

We recently developed a genetic mouse model where endogenous GDNF expression is increased at the post-transcriptional level via 3′UTR editing. This approach warrants expression in cells that transcribe GDNF naturally (Kumar et al., 2015). We found that constitutive elevation in endogenous GDNF levels protects dopaminergic neurons in the proteasome inhibitor lactacystin (LC) induced PD model (Kumar et al., 2015). However, because GDNF is involved in dopamine neuron development (Lin et al., 1993; Burke, 2003; Oo, 2003), this model does not allow to address whether GDNF upregulation is beneficial because of developmental effects, and whether similar protection could be achieved in adults. Therefore, to evaluate the therapeutic potential of endogenous GDNF, it is imperative to use a system that allows the analysis of conditional, adult-onset increase in endogenous GDNF expression.

To that end, we developed GDNF conditional hypermorphic (*Gdnf*^cHyper^) mice (Mätlik et al., 2022). We analyzed the therapeutic potential of endogenous GDNF upregulation in the striatum in the LC-induced model of PD, using both neuroprotection and neurorestoration paradigms. We measured both motor and nonmotor symptoms with a variety of behavioral tests and assessed brain dopamine levels and the number of dopamine neurons. Our results show that upregulation of endogenous GDNF in the adult striatum is not protective or neurorestorative in LC-induced PD model. Since aging is the highest risk factor for PD, we also analyzed the effect of endogenous GDNF deletion in aged GDNF conditional knock-out mice. We found that brain-specific GDNF deletion does not increase susceptibility of the dopamine system to LC-induced damage. Altogether, our results show that neither a 2–4-fold increase or deletion of GDNF impact the outcome in the LC-induced proteasome inhibition mouse model of PD.

## Materials and Methods

### Animals

**Figure 1.**
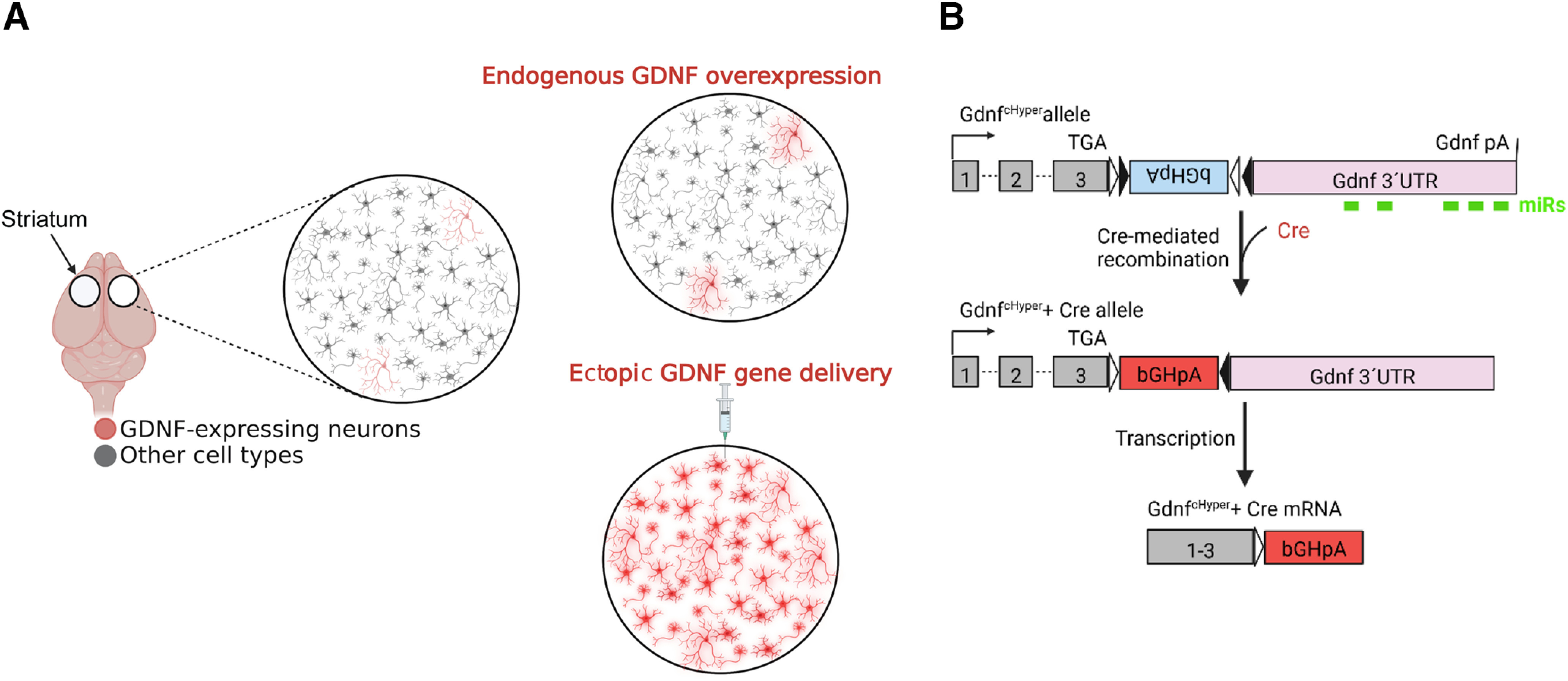
Schematic of endogenous GDNF overexpression. ***A***, Comparison between endogenous GDNF overexpression and ectopic *Gdnf* delivery. ***B***, Schematic of the *Gdnf^cHyper^* allele. A FLEx cassette containing loxP sites flanking a short 3′UTR that includes the bovine growth hormone polyadenylation sequence (bGHpA) in an inverted orientation was inserted immediately downstream of *Gdnf* stop codon (top panel). Upon Cre-mediated recombination, the FLEx cassette is inverted (middle panel) and the resulting Gdnf transcript contains a short 3′UTR devoid of microRNA (miR) sites (lower panel).

The study was performed on 129Ola/ICR/C57bl6 mixed background male mice. The mice were provided with *ad libitum* access to food and water at temperature-controlled conditions at 20–22°C under 12/12 h light/dark cycle at a relative humidity of 50–60%. Bedding material and cages (aspen chips, Tapvei Oy) were changed weekly along with wooden tube and aspen shavings as enrichment. Generation and genotyping of *Gdnf*-floxed and GDNF conditional hypermorphic (cHyper) mice were described previously (Kopra et al., 2015; Mätlik et al., 2022). A schematic of the *Gdnf^cHyper^* allele is shown on Fig. 1. All animal experiments were conducted considering the 3R principles of the EU directive 2010/63/EU governing the care and use of experimental animals and authorized by the County Administrative Board of Southern Finland (license numbers ESAVI-2010–09011/Ym-23 and ESAVI/11 198/04.10.07/2014). All the protocols were authorized by the National Animal Experiment Board of Finland.

### Experimental design

The study consists of five experiments, named I through V. The details of the experimental procedures are described separately for each experiment. In all experiments, baseline behavioral tests were performed before the injections. In all experiments, we used male mice. Sample size was determined based on previous studies using male mice with the same triple-mixed genetic background. No statistical methods were used to predetermine sample size. Only litters including animals from different genotypes were included. Animals were tested in a random order. Stereotaxic injections were administered in a random order. Tissue isolations were performed in a random order. All experiments were performed by researchers blinded to the genotypes of the animals.

**Figure 2.**
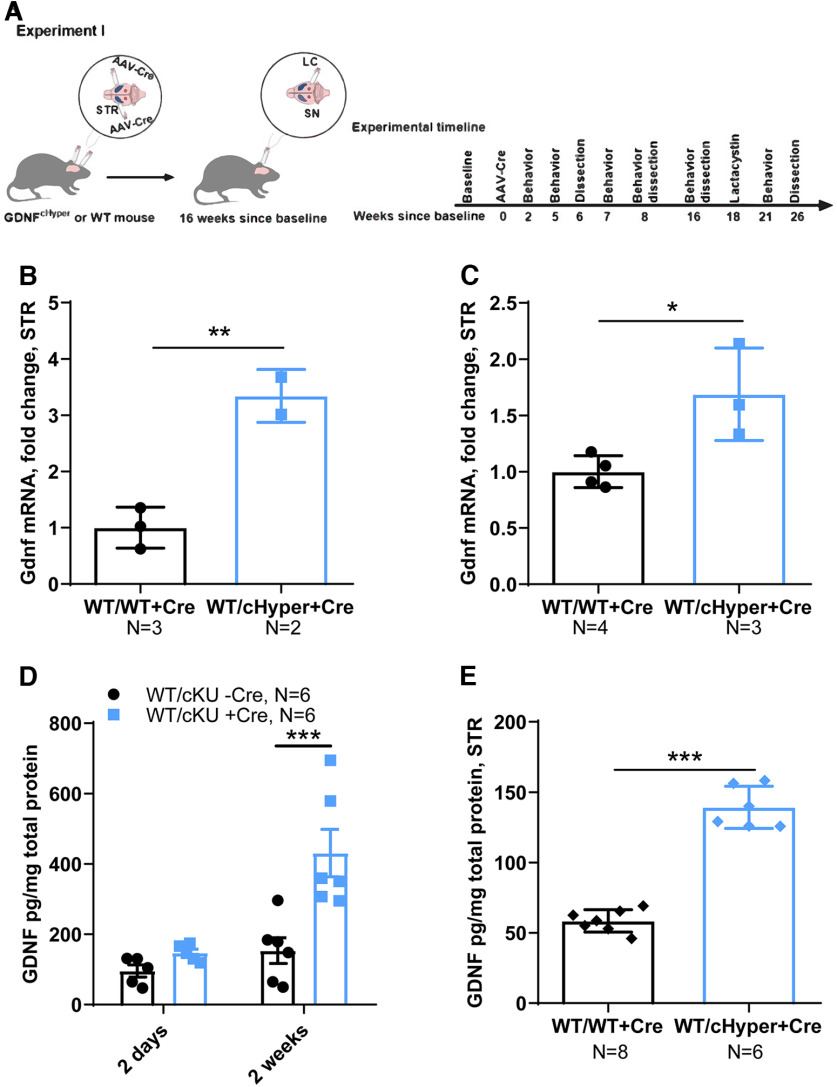
Analysis of endogenous GDNF mRNA and protein expression in the striatum after striatal AAV5-Cre delivery. ***A***, Schematic of experimental design to evaluate GDNF expression levels after striatal AAV-Cre delivery in *Gdnf^cHyper^* mice. ***B***, Striatal Gdnf mRNA levels measured with qPCR six weeks after bilateral AAV-Cre injection into the STR in *Gdnf^cHyper^* mice. Unpaired *t* test. *p* = 0.0078. ***C***, Striatal Gdnf mRNA levels 26 weeks after bilateral AAV-Cre injection into STR, measured with qPCR. Unpaired *t* test. *p* = 0.02. ***D***, Striatal GDNF protein levels in *Gdnf^cHyper^* mice 2 d and two weeks after striatal AAV-Cre injection, measured with ELISA. Two-way repeated measures ANOVA, Sidak’s multiple comparisons test. *p* = 0.0003. ***E***, GDNF protein levels in *Gdnf^cHyper^* mice striatum eight weeks after baseline, measured with ELISA. Unpaired *t* test. *p* = 0.0001.

In Experiment I (Fig. 2*A–E*), adult *Gdnf^w/wt^* and *Gdnf^wt/cHyper^* mice were injected bilaterally with AAV5-Cre into the striatum to induce GDNF upregulation. Mice were dissected at two days, and two, six, and eight weeks after the AAV5-Cre injection to measure mRNA and protein levels.

**Figure 3.**
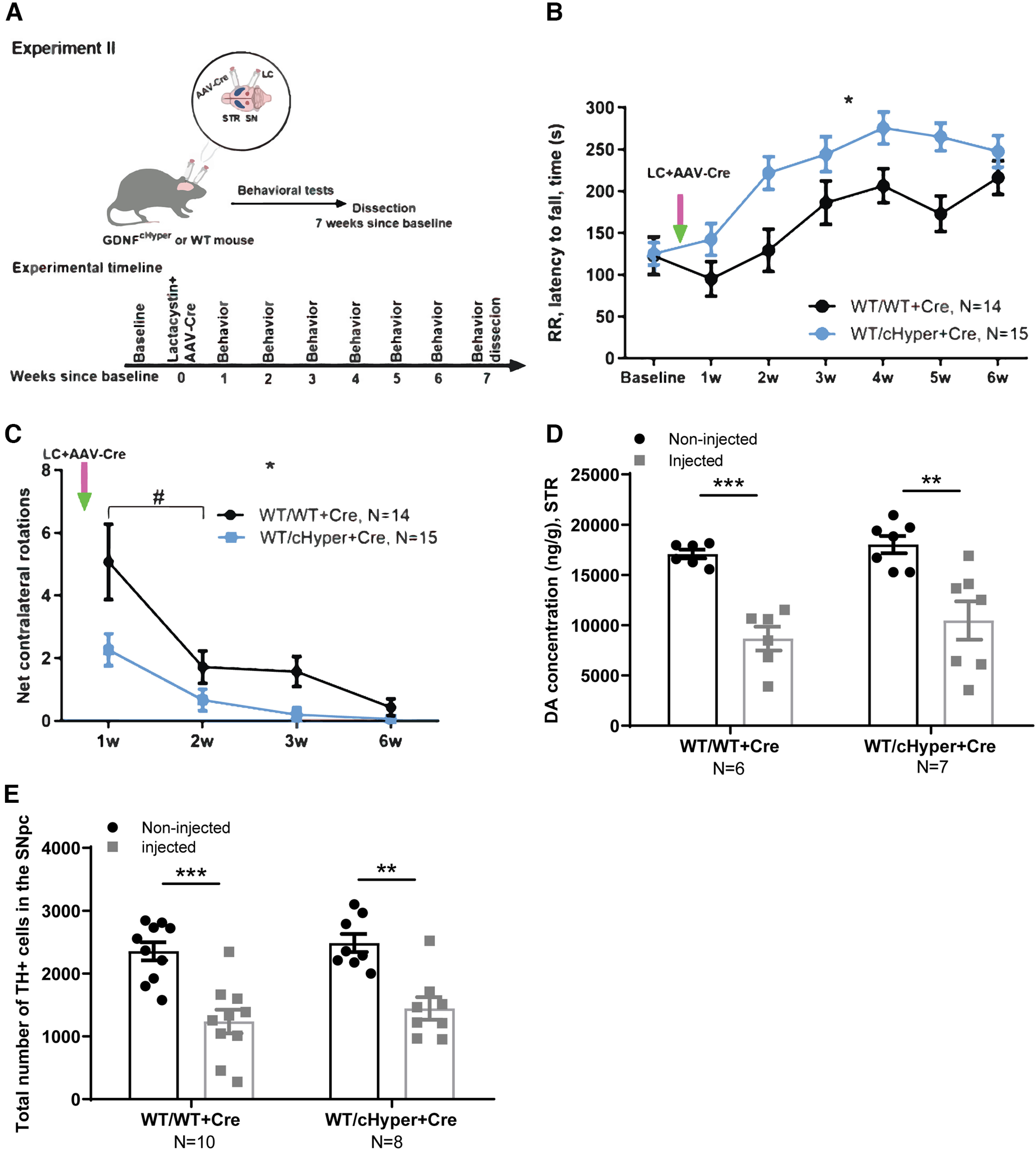
Assessment of GDNF effects with simultaneous LC injection in *Gdnf^cHyper^* mice. ***A***, Schematic of experiment for AAV-Cre and LC simultaneous injection. ***B***, Latency to fall in the accelerating rotarod test. Two-way repeated measures ANOVA, Sidak’s multiple comparisons test. *p* = 0.0108 genotype effect. ***C***, Number of contralateral rotations one, two, three, and six weeks after simultaneous LC and AAV-Cre injections. Two-way repeated measures ANOVA, Sidak’s multiple comparisons test. *p* = 0.0103 genotype effect. #*p* < 0.0001 (WT/WT+Cre); *p* = 0.05 (WT/cHyper+Cre) between one week and two weeks after LC and AAV-Cre injection. ***D***, Total tissue dopamine levels in the injected and noninjected STR, measured with HPLC. Two-way repeated measures ANOVA, Tukey’s multiple comparisons test. *p* = 0.0009; *p* = 0.0013. ***E***, The number of TH-positive cells indicating the number of dopaminergic neurons in the SNpc. Two-way repeated measures ANOVA, Tukey’s multiple comparisons test. *p* = 0.0001; *p* = 0.0013 between injected and noninjected side. LC, lactacystin; cHyper, conditional hypermorphic; WT, wild type; STR, striatum; SNpc, substantia nigra pars compacta; RR, rotarod; CR, contralateral rotations; DA, dopamine; TH, tyrosine hydroxylase. *p < 0.05, **p < 0.01, ***p < 0.001. See Extended Data Figure 3-1 for cylinder test and body weight data.

In Experiment II (Fig. 3*A–E*), adult *Gdnf^w/wt^* and *Gdnf^wt/cHyper^* mice were injected unilaterally with LC above the right SN to induce dopaminergic cell death, and simultaneously with AAV5-Cre into the right striatum to induce GDNF upregulation. The noninjected (left) hemisphere was considered a control. The animals were followed with indicated behavioral tests and were dissected seven weeks after the injections.

10.1523/ENEURO.0097-22.2023.f3-1 Extended Data Figure 3-1 Related to Figure 3. Elevation of GDNF expression levels after simultaneous AAV-Cre and LC injection and the effects on cylinder test and body weight. ***A***, Ratio of left forepaw use in the cylinder test one, two, three, and six weeks after LC and AAV-Cre simultaneous injection. Two-way repeated measures ANOVA, Sidak’s multiple comparisons test. ***B***, Body weight from the baseline until the end of the experiment. Two-way repeated measures ANOVA, Sidak’s multiple comparisons test. LC, lactacystin; WT, wild type; cHyper, conditional hypermorphic; STR, striatum; Cyl, cylinder test; BW, body weight. *p < 0.05, **p < 0.01, ***p < 0.001. Download Figure 3-1, TIF file.

**Figure 4.**
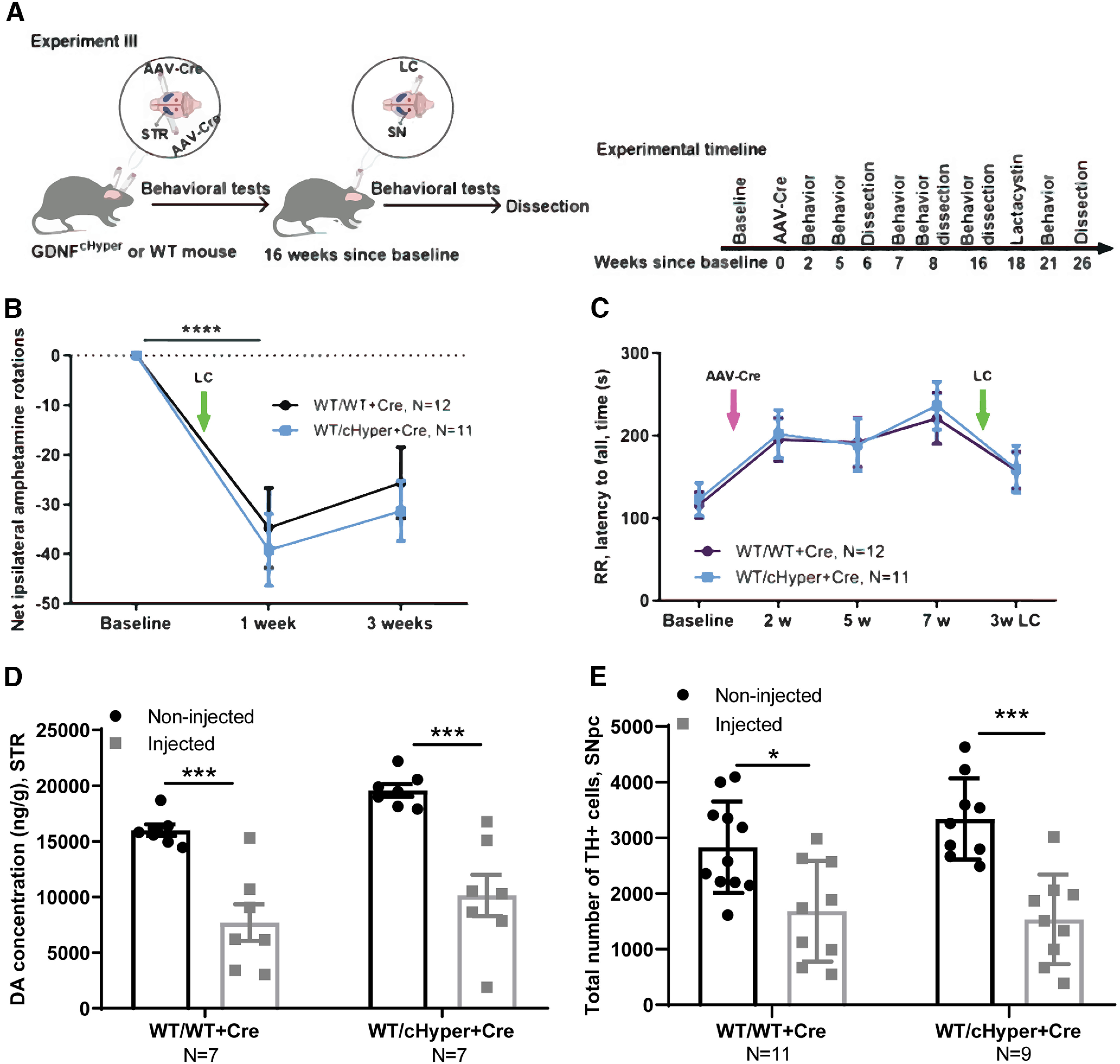
Neuroprotection analysis, *Gdnf^cHyper^* mice injected with AAV-Cre into the striatum, followed by LC injection 16 weeks later. ***A***, Schematic of experiment for AAV-Cre bilateral injection into the STR and unilateral LC injection above the SN 16 weeks later. ***B***, The number of contralateral rotations after intraperitoneal amphetamine administration one and three weeks after LC. Two-way repeated measures ANOVA, Sidak’s multiple comparisons test. *p* < 0.0001 significant difference in both genotypes compared with the baseline. ***C***, Latency to fall in accelerating rotarod test two, five, seven, and 21 weeks after AAV-Cre injection. Two-way repeated measures ANOVA, Sidak’s multiple comparisons test. ***D***, Total tissue dopamine levels in the STR, measured with HPLC. Two-way repeated measures ANOVA, Tukey’s multiple comparisons test. *p* = 0.0008 (WT/WT+Cre); *p* = 0.0002 (WT/cHyper+Cre) between injected and noninjected side in both genotypes. ***E***, The number of TH-positive cells indicating the number of dopaminergic neurons in the SNpc. Two-way repeated measures ANOVA, Tukey’s multiple comparisons test. *p* = 0.0180; *p* = 0.0002 between injected and noninjected side. LC, lactacystin; cHyper, conditional hypermorphic; WT, wild type; STR, striatum; SNpc, substantia nigra pars compacta; AR, amphetamine rotations; RR, rotarod; DA, dopamine; TH, tyrosine hydroxylase. *p < 0.05, **p < 0.01, ***p < 0.001. See Extended Data Figure 4-1 for cylinder test data.

In Experiment III (Fig. 4*A–E*), adult *Gdnf^w/wt^* and *Gdnf^wt/cHyper^* mice were injected bilaterally with AAV5-Cre into the striatum to induce GDNF upregulation. The mice were followed with indicated behavioral tests. At 16 weeks after the AAV-Cre injections, a subset of mice were unilaterally injected with LC above the right substantia nigra, followed by behavioral tests. The animals were dissected eight weeks after LC injection.

**Figure 5.**
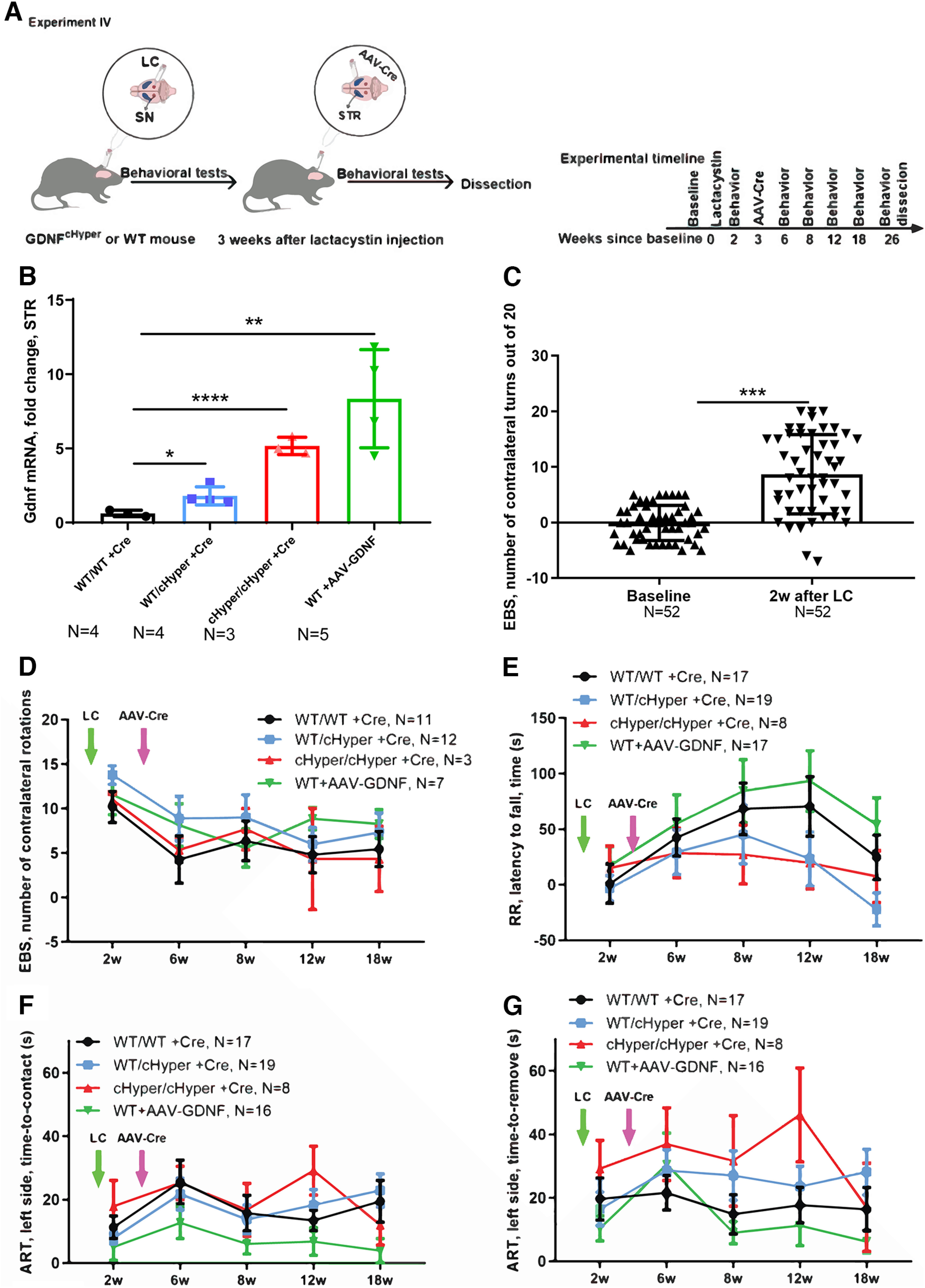
Neurorestoration analysis – *Gdnf^cHyper^* and WT mice injected with LC followed by AAV-Cre and AAV5-GDNF injection three weeks later. ***A***, Schematic of experiment depicting unilateral injections of LC just above the SN and AAV-Cre or AAV5-GDNF into the STR three weeks later. ***B***, Striatal Gdnf mRNA levels measured with qPCR at the endpoint (26W). One-way ANOVA, Tukey’s multiple comparisons test. *p* = 0.0203, *p* = 0.0001, *p* < 0.0001. ***C***, Number of net contralateral turns (contralateral minus ipsilateral) in the elevated body swing test two weeks after LC injection (out of 20 turns). Unpaired *t* test. *p* = 0.0001. ***D***, Number of contralateral turns (left-biased turns) in the elevated body swing test after LC and AAV-Cre injections. Two-way repeated measures ANOVA, Tukey’s multiple comparisons test. ***E***, Latency to fall in the accelerating rotarod test. Two-way repeated measures ANOVA, Tukey’s multiple comparisons test. ***F***, Time to touch the adhesive in the left forepaw in the adhesive removal test. Two-way repeated measures ANOVA, Tukey’s multiple comparisons test. ***G***, Time to remove the adhesive from the left forepaw in the adhesive removal test. Two-way repeated measures ANOVA, Tukey’s multiple comparisons test. LC, lactacystin; cHyper, conditional hypermorphic; WT, wild type; STR, striatum; SN, substantia nigra; EBS, elevated body swing; RR, rotarod; ART, adhesive removal test. *p < 0.05, **p < 0.01, ***p < 0.001. see Extended Data Figure 5-1 for open field, home-cage activity, nesting score, and body weight data and Extended Data Figure 5-2 for IBA1 and GFAP immunohistochemistry data.

**Figure 6.**
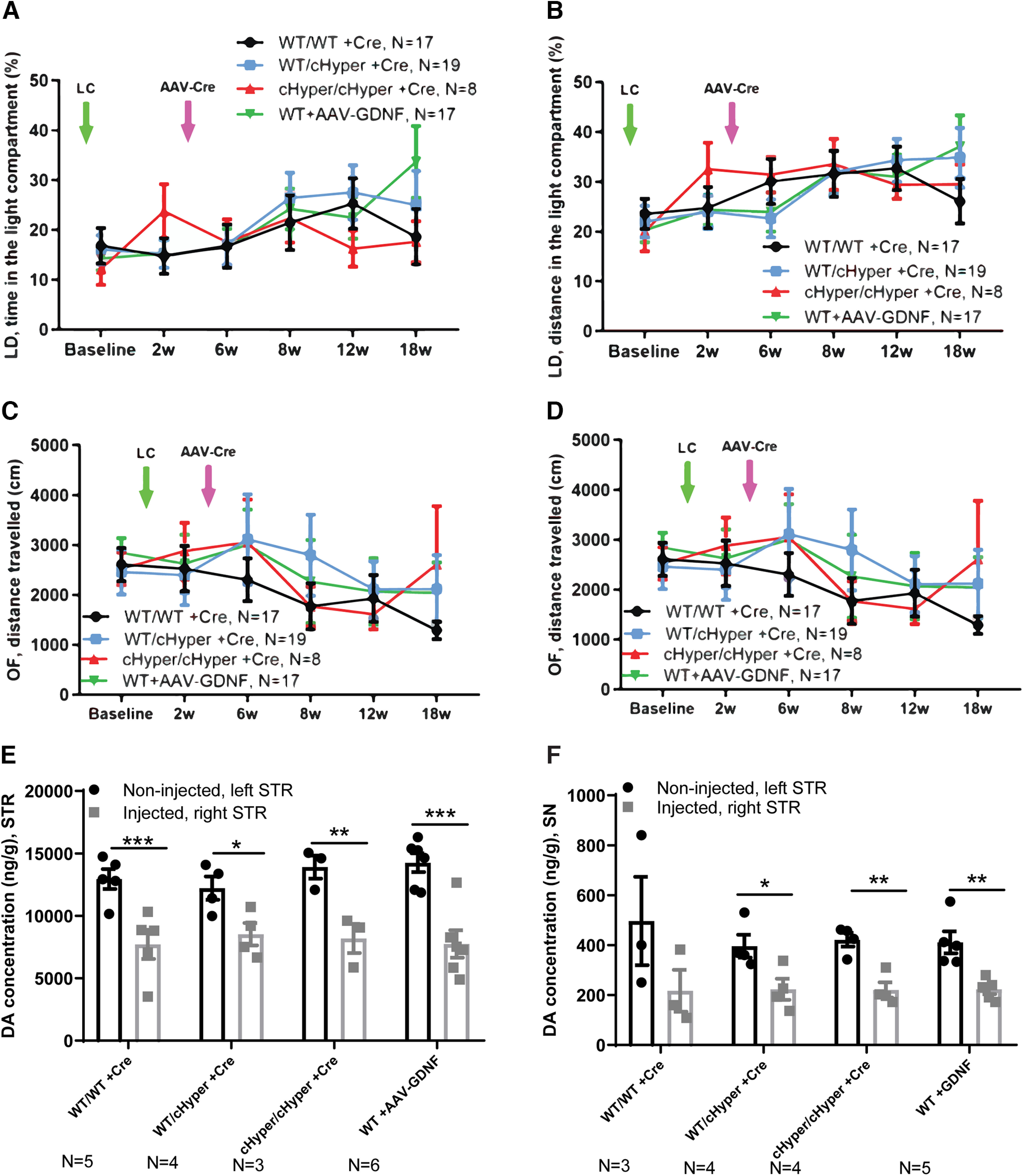
Neurorestoration analysis: *Gdnf^cHyper^* and WT mice injected with LC, followed by AAV-Cre and AAV5-GDNF injection three weeks later (continues from Fig. 3). ***A***, Time spent in the light compartment in the light-dark test. Two-way repeated measures ANOVA, Tukey’s multiple comparisons test. ***B***, Distance traveled in the light compartment in the light-dark test. Two-way repeated measures ANOVA, Tukey’s multiple comparisons test. ***C***, Total distance traveled in the open field over 30 min. Two-way repeated measures ANOVA, Tukey’s multiple comparisons test. ***D***, Number of rearings indicating vertical activity in the open field. Two-way repeated measures ANOVA, Tukey’s multiple comparisons test. ***E***, Total tissue dopamine levels in the STR, measured with HPLC. Two-way repeated measures ANOVA, Tukey’s multiple comparisons test. *p* = 0.0005, *p* = 0.02, *p* = 0.002, *p* = 0.00001 between injected and noninjected side. ***F***, Total tissue dopamine levels in the SN, measured with HPLC. Two-way repeated measures ANOVA, Tukey’s multiple comparisons test. *p* = 0.03, *p* = 0.002, *p* = 0.004 between injected and noninjected side. LC, lactacystin; cHyper, conditional hypermorphic; WT, wild type; LD, light-dark; OF, open filed; STR, striatum; SN, substantia nigra; DA, dopamine. *p < 0.05, **p < 0.01, ***p < 0.001.

In Experiment IV (Figs. 5*A–G*, 6*A–F*), *Gdnf^w/wt^*,*Gdnf^wt/cHyper^* and *Gdnf^cHyper/cHyper^* mice were injected unilaterally with LC above the right substantia nigra to induce dopaminergic cell death and followed with behavioral tests as indicated in Figures 5 and 6. To induce GDNF upregulation, AAV5-Cre was injected unilaterally into the right striatum three weeks after LC injection. As a control, we also injected wild-type mice with AAV5-GDNF to compare the outcomes after ectopic and endogenous GDNF overexpression. The animals were followed in behavioral tests and were dissected 26 weeks after baseline.

**Figure 7.**
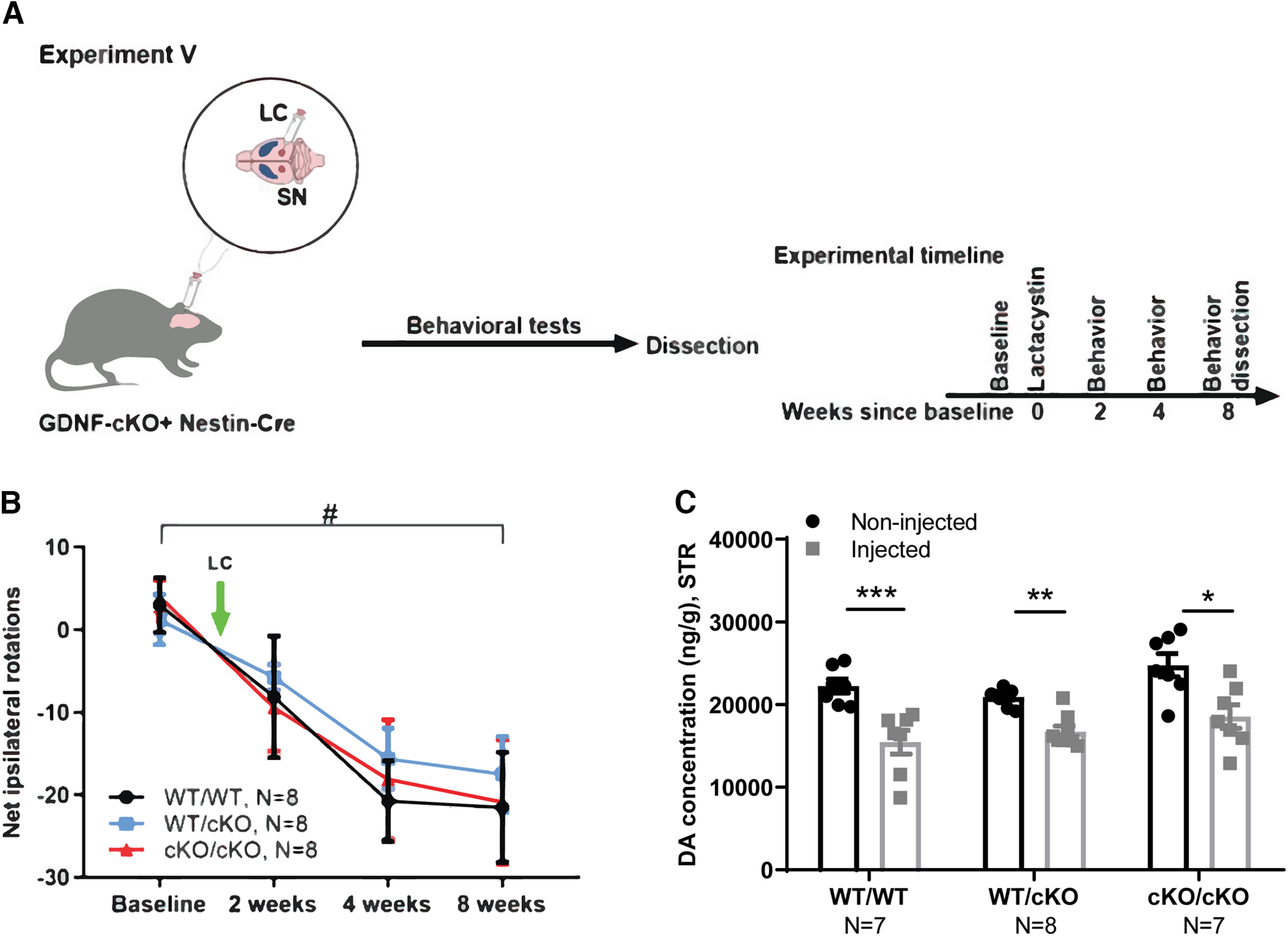
Neuroprotection analysis, removal of endogenous GDNF using conditional knock-out (cKO) allele. ***A***, Schematic of experiment for *Gdnf^cKO^*;Nestin-Cre mice injected with LC just above the right SN. ***B***, The number of contralateral rotations, indicating the severity of the lesion after intraperitoneal amphetamine administration two, four, and eight weeks after LC injection. Two-way repeated measures ANOVA, Tukey’s multiple comparisons test. #*p* = 0.002 (WT/WT+Cre), *p* = 0.03 (WT/cKO+Cre), *p* = 0.005 (cKO/cKO+Cre) between baseline and eight weeks after LC injection. ***C***, Total tissue dopamine levels in the STR at the endpoint (8w), measured with HPLC. Two-way repeated measures ANOVA, Tukey’s multiple comparisons test. LC, lactacystin; WT, wild type; SN, substantia nigra; STR, striatum; AR, amphetamine rotation; DA, dopamine. *p < 0.05, **p < 0.01, ***p < 0.001.

In Experiment V (Fig. 7*A–C*), *Gdnf^cKO^* mice were crossed with the Nestin-Cre line (Kopra et al., 2015) to delete GDNF specifically in the CNS. Thirteen- to 15-month-old male mice were unilaterally injected with LC just above the right substantia nigra. Mice were followed with behavioral tests as indicated on Figure 4 and were dissected eight weeks after LC injection.

### Behavioral tests

Behavioral tests including rotarod (RR), open field (OF), and light-dark exploration (LD) were performed as previously described (Mätlik et al., 2018).

### Amphetamine-induced rotations

Animals were injected with 1–4 mg/kg amphetamine, i.p. and placed into a bucket. Rotations were recorded starting immediately after amphetamine injection and were recorded for 40 min. Rotations in the ipsilateral direction were counted starting from 10 min after the injection. In Experiments I–IV, mice were injected with 1 mg/kg amphetamine. In Experiment V, mice were injected with 4 mg/kg amphetamine, i.p., at baseline and with 2 mg/kg amphetamine, i.p., in subsequent experiments.

### Elevated body swing test

Elevated body swing test (EBS) was performed to evaluate asymmetrical motor behavior (Borlongan and Sanberg, 1995). The mouse was held by the base of the tail and lifted 20 times, allowing it to briefly rest after each trial. A swing was counted on the movement of the head in either direction.

### Cylinder test

Cylinder test was performed to evaluate motor asymmetry (Su et al., 2018). Mice were placed into a 20-cm diameter glass cylinder for 5 min, and the number of first and total touches with each forepaw on the cylinder wall were counted.

### Spontaneous rotations

Spontaneous rotations were counted to measure motor asymmetry. Mice were placed into a 20-cm diameter glass cylinder for 5 min, and the number of full ipsilateral and contralateral rotations (360°) were counted.

### Home cage activity

Spontaneous activity in a home cage setting was measured using the TSE InfraMot system (TSE). The mice were single housed with nesting material and *ad libitum* access to water and food. Circadian activity was recorded during a period of 8 d. For the analysis, activity measures were divided into 6-h blocks, 12/12 h light/dark periods, and total circadian activity over 24 h. On the last day of the home-cage activity test, the nesting was scored according to a five-point scale (Deacon, 2006).

### Adhesive removal test

Adhesive removal test was performed to evaluate sensorimotor deficits. Mice were habituated in the testing cage for 60 s, and a small adhesive tape was adhered to the underside of each forepaw. The mouse was placed back in the testing cage and the time to touch and remove the tape were recorded for each forepaw separately. The cutoff time was set at 2 min.

### Stereotaxic surgery

Mice were anesthetized with isoflurane (4% for induction, 1.5–2% for maintenance) in 100% O_2_. The hair on the top of the head was shaved and cleaned with Desinfektol P (Berner Pro). Mice were placed on a stereotaxic apparatus and lidocaine (Yliopiston Apteekki) was injected into the skin for local analgesia. The skin was cut with a scalpel and holes were drilled into the skull.

LC injections were performed using a 10-μl Hamilton syringe with a 26-G steel needle (A.G. Scientific/Nordic Biosite). The needle was unilaterally inserted into the right hemisphere of the brain just above SN (A/P −3.3; M/L −1.2 and D/V −4.6, relative to bregma). For all the experiments (Experiments I–V), we used 2 μg LC in 1 μl of DPBS.

AAV5-Cre and AAV5-GDNF injections were performed as described previously (Mätlik et al., 2022). Briefly, a 33-G blunt NanoFil needle (World Precision Instruments) was inserted into the brain parenchyma at a 10° angle to avoid damaging the lateral ventricle. AAV5-Cre was injected at A/P 1.2 and 0.2; M/L ±2.2 and D/V −3.0, relative to bregma. In Experiments I and III, injections were performed bilaterally, and in Experiments II and IV, injections were performed unilaterally into the right striatum.

The mice received 5 mg/kg Rimadyl intraperitoneally (Yliopiston Apteekki) after the surgery for postoperative analgesia. Mortality in each experiment was as follows: I and III, 0%; II, 9.4%; IV, 21%; V, 3.7%.

### Tissue isolation

Mice were dissected at different time points as described above in Experimental design. Striatum and substantia nigra (SN) were dissected using a standard mouse brain matrix and were snap-frozen on dry ice immediately for gene expression, protein levels, and high-performance liquid chromatography (HPLC) analyses. For histologic analyses, tissues were under light perfusion with PBS which allows us to analyze STR for DA and SN pars compacta (SNpc) for tyrosine hydroxylase (TH)-positive cells from the same animal. Tissues for immunohistochemistry were fixed o/n in 4% formaldehyde for stereological analyses of TH-positive cells in the SNpc.

### High-performance liquid chromatography (HPLC)

Dopamine levels were measured in striatum and SN brain samples as previously described (Valros et al., 2015), using HPLC with electrochemical detection.

### Stereological analysis of TH-positive cells

The number of TH-positive neurons was assessed in SNpc as described previously (Kumar et al., 2015). TH-positive cells were counted at the medial region of SNpc. StereoInvestigator (MBF Bioscience) was used to outline the SNpc and positively stained cells were counted within the defined outlines.

### Immunohistochemistry of IBA1 and GFAP in SN

Immunohistochemistry was performed on freely floating 40-μm coronal cryosections, stored at −20°C in cryopreservant containing 30% ethylene glycol and 20% glycerol in 0.1 m phosphate buffer. IBA1 and GFAP stains were performed on separately on different sections containing the substantia nigra from the same animals. The primary antibodies used were an anti-GFAP antibody raised in rabbit (Fisher, RB-087-A0) and an anti-IBA1 antibody raised in rabbit (Wako, 019-19741) visualized with an AlexaFluor 594-conjugated donkey anti-rabbit secondary antibody (Abcam, ab150064).

### Image analysis

Stained mouse SN containing sections from three to four male mice per experimental group (approximately −3.08 to −3.64 mm AP from bregma, according to the brain atlas from Franklin and Paxinos, 2013) were imaged at 20× magnification using a Zeiss Axio Imager microscope outfitted with a Zeiss ApoTome for optical sectioning. Eleven successive fluorescence images were taken every 0.5 μm using the ApoTome optically sectioned with structured illumination to produce a 6.5-μm-thick 3-D final image of both the injected and noninjected substantia nigra. Only sections in which the injection injury was readily identifiable were used. Images were analyzed using Imaris software to quantify the overall density within the substantia nigra of the stained protein of interest (calculated as a ratio defined as total volume of protein stain/total volume of image) by a researcher blind to experimental condition. The ratio of the stain density from the injected side was then taken relative to the stain density of the noninjected side of one to three slices and averaged for each individual animal.

### Gene expression analyses

RNA was isolated using TRIzol reagent (Thermo Fisher Scientific) according to the manufacturer’s protocol. cDNA was synthesized from 500 ng of total DNaseI-treated RNA using random hexamer primers and RevertAid Reverse Transcriptase (Thermo Fisher Scientific). Real-time quantitative PCR (qPCR) was conducted with the LightCycler 480 instrument using SYBR Green I Master (Roche Diagnostics). PCR reaction was run in duplicate in a total volume of 10 μL containing 0.25 mm primers and cDNA equivalent to 0.25 ng of RNA in a 384-well plate. Mouse *Actb* was used as a reference gene.

The quantification cycle (C_q_) values were obtained from LightCycler 480 Software Release 1.5.0 SP1 using the Absolute Quantification/second Derivative Max option. Gdnf relative expression was analyzed as described previously (Varendi et al., 2014). Primer sequences were as follows: β-actin-F: CTAAGGCCAACCGTGAAAAG, β-actin-R:ACCAGAGGCATACAGGGACA, Gdnf-F: CGCTGACCAGTGACTCCAATATGC and Gdnf-R: TGCCGCTTGTTTATCTGGTGACC.

### Analysis of protein levels

GDNF Emax Immunoassay System (G7620, Promega) was used to measure GDNF protein levels, according to the manufacturer’s protocol. Mouse striatum samples were homogenized in a lysis buffer recommended by the manufacturer and the homogenate was centrifuged at 5000 rpm for 15 min at 4°C. The supernatant was acid-treated using HCl, and GDNF protein levels and total protein level were measured and analyzed as described previously previously (Kumar et al., 2015). Striatal samples from *Gdnf^cKO^* x Nestin-Cre mice were used as control.

### Quantification and statistical analysis

All values are shown as mean ± SEM. Research subjects correspond to measurements from individual animals. Statistical comparisons were performed using an unpaired Student’s *t* test with two-tailed distribution, or one-way or two-way ANOVA, followed by Tukey’s or Sidak’s *post hoc* test, where appropriate. qPCR data were analyzed as described previously (Varendi et al., 2014). The level of statistical significance was set at *p* < 0.05.

## Results

### Analysis of GDNF expression dynamics in *Gdnf^cHyper^* mice

The principle of *Gdnf cHyper* allele and the effect of conditional 3′UTR replacement on Gdnf expression are shown in Figure 1. First, to evaluate the extent and timeline of endogenous GDNF upregulation in the adult striatum, we performed bilateral AAV-Cre injections into the striatum of *Gdnf^wt/Hyper^* mice (Experiment I; Fig. 2*A*) and analyzed striatal Gdnf mRNA and protein levels at different time points (Fig. 2*B–E*). We found that Gdnf mRNA levels were upregulated in striatum of *Gdnf^wt/cHyper^* +Cre compared with control mice six and 26 weeks after AAV-Cre injection (Fig. 2*B*,*C*). We subsequently measured GDNF protein levels 2 d and two and eight weeks after AAV-Cre injection and found that GDNF protein levels were ∼2–3-fold increased two and eight weeks after AAV-Cre delivery (Fig. 2*D*,*E*).

### Elevation of endogenous GDNF expression transiently improves motor function but does not protect dopamine system if induced simultaneously with LC injection

To gain insight into how endogenous GDNF levels modulate dopaminergic function on inhibition of proteasome function via supranigral LC delivery, we performed unilateral AAV-Cre injection into the right striatum, followed by LC injection into the right SN on the same day (Experiment II; Fig. 3*A*). The mice were monitored over a period of seven weeks using the accelerating rotarod and cylinder tests to evaluate motor function and the extent of unilateral damage. Baseline tests were performed one week before injection. We found that in the accelerating rotarod test, *Gdnf^wt/Hyper^* mice performed significantly better compared with wild-type littermates starting from one week after AAV-Cre/LC injection (Fig. 3*B*).

Since LC induced unilateral damage, we also evaluated the number of spontaneous contralateral rotations and found that the number of rotations was significantly smaller in *Gdnf^wt/cHyper^* mice compared with controls (Fig. 3*C*), suggesting a possible reduction in the extent of SN damage. However, the ratio of left-to-total (right hemisphere of the brain was injected) number of forepaw touches in the cylinder test revealed no differences between the genotypes (Extended Data Fig. 3-1*A*). We did not observe a significant difference in body weight (Extended Data Fig. 3-1*B*).

Next, we asked how endogenous GDNF upregulation in the striatum affects dopamine levels in LC-injected mice. We found that while LC injection significantly reduced dopamine levels in both genotypes, there were no significant differences between the genotypes (Fig. 3*D*). In line with these findings, the number of TH+ cells in the SNpc after LC injection was reduced by ∼50% in both groups, but there was no difference between the genotypes (Fig. 3*E*). Altogether, these results indicated that upregulation of endogenous GDNF, when induced simultaneously with LC injection, may transiently improve motor function, but does not preserve dopamine levels in the striatum or TH+ cells in the SNpc.

### Analysis of endogenous GDNF upregulation in neuroprotection paradigm in LC induced PD model

The above results suggested that endogenous GDNF upregulation does not protect dopaminergic neurons in a LC model when induced simultaneously with LC delivery. We next asked whether elevation of endogenous GDNF could be neuroprotective if induced before LC delivery and cell death. To address this, we injected AAV-Cre bilaterally into the striatum of *Gdnf^wt/cHyper^* and *Gdnf*w/w mice, and injected LC into the right SN 16 weeks later (Fig. 4*A*). First, to study the degree of unilateral damage, we quantified amphetamine-induced rotations one and three weeks after LC injection. Amphetamine-induced rotations reflect the difference in dopamine release between the lesioned and the nonlesioned striata. This test is commonly used to monitor the extent of motor impairment induced by unilateral lesion in the dopaminergic system (Björklund and Dunnett, 2019). We observed a significant reduction in the number of net ipsilateral rotations one week after LC injections, demonstrating a unilateral lesion, but there were no significant differences between the genotypes (Fig. 4*B*). Similarly, the accelerating rotarod test did not reveal any differences between the genotypes neither at baseline, nor after LC injection (Fig. 4*C*). Moreover, the number of touches in the cylinder test was not different between the genotypes (Extended Data Fig. 4-1). In line with the previous experiment, there was a significant reduction in striatal dopamine levels and TH+ cells in the SNpc in both groups, but no differences between the genotypes (Fig. 4*D*,*E*). Collectively, these data demonstrate that a 2–3-fold upregulation of endogenous GDNF in the striatum is not neuroprotective in a LC-induced PD model.

10.1523/ENEURO.0097-22.2023.f4-1 Extended Data Figure 4-1 Related to Figure 4. Analysis of motor behavior in neuroprotection paradigm. Number of first touches in the cylinder test made with either right, left or both forepaws (3 weeks after LC). WT, wild type; cHyper, conditional hypermorphic; STR, striatum; Cyl, cylinder test. Download Figure 4-1, TIF file.

### Analysis of endogenous GDNF upregulation in neurorestoration paradigm in LC induced PD model for motor and nonmotor symptoms

Our results suggested that elevation of endogenous GDNF is not neuroprotective in a LC-induced PD model. However, since earlier studies have shown that ectopic GDNF promotes dopamine synthesis and turnover in cultured dopaminergic neurons and induces TH+ fiber outgrowth *in vivo* (Lin et al., 1993; Hoffer et al., 1994), we asked whether endogenous GDNF could be neurorestorative if given at a time when substantial LC-induced neurodegeneration has already occurred. To address this possibility, we first performed unilateral LC injections into the SN and subsequently injected the mice with AAV-Cre or AAV5-GDNF to in parallel assess the effect of ectopic GDNF expression into the right striatum three weeks later (Experiment IV; Fig. 5*A*). In addition, to address the possibility that the increase in endogenous GDNF in heterozygous *Gdnf^wt/cHyper^* mice was not sufficient to confer neuroprotection and/or neurorestoration, we included the homozygous *Gdnf^cHyper/cHyper^* mice in this experiment. At the endpoint of 26 weeks after LC injection, Gdnf mRNA levels were allele-dose-dependently increased in the striatum of *Gdnf^cHyper^* mice (Fig. 5*B*). Mice were analyzed with a comprehensive set of behavioral tests commonly used to evaluate both the motor and nonmotor symptoms in PD models. To evaluate the extent of unilateral motor damage, we used the elevated body swing test and found that LC significantly increased the swing activity to the direction contralateral to the lesioned side two weeks after the injections (Fig. 5*C*). However, there were no differences between the genotypes at any of the time points tested (Fig. 5*D*). Similarly, the accelerating rotarod test revealed no significant difference in motor behavior of the mice between the groups (Fig. 5*E*). When sensorimotor deficits were evaluated with the adhesive removal test, we found no differences in the time to contact or the time to remove the adhesive from the paw at any of the tested time points (Fig. 5*F*,*G*). For motor symptoms, the open field test did not reveal differences in the distance traveled, vertical activity, average velocity, or the resting time between the genotypes (Fig. 6*C*,*D*; Extended Data Fig. 5-1*A*,*B*,*D*). We then performed the light-dark test to evaluate anxiety-related behavior but did not observe differences in the time and distance traveled in the light compartment (Fig. 6*A*,*B*). Similarly, anxiety-related behavior was unaltered as evaluated by time spent in the center in the open field test (Extended Data Fig. 5-1*C*). We also did not observe differences in spontaneous activity during light and dark period in the home cage activity test (Extended Data Fig. 5-1*E*,*F*), or in nesting behavior (Extended Data Fig. 5-1*G*), altogether suggesting no change between genotypes for nonmotor symptoms. Finally, at the endpoint of 26 weeks after LC injection, the mice were analyzed for brain dopamine levels in the striatum and SN. As in previous experiments, although LC treatment significantly reduced dopamine levels in the striatum and SN in all genotypes, there were no significant differences between the genotypes (Fig. 6*E*,*F*). Moreover, while there was a clear trend toward increased IBA1 immunoreactivity in WT animals on the injected side, we did not see any changes in GFAP (Extended Data Fig. 5-2*A–D*). This is in line with previous work which similarly reported a significant increase in IBA1 and a trend towards increased GFAP immunoreactivity in the SN in LC PD model (Savolainen et al., 2017). Similarly, research from another study focusing on neuroinflammation, through acute or repeated LPS injection found changes in IBA1 immunoreactivity but not GFAP immunoreactivity (Norden et al., 2016). Altogether, Experiments II–IV show that AAV-Cre-induced upregulation of endogenous GDNF in the adult striatum is not neuroprotective or neurorestorative in a LC-induced PD model.

10.1523/ENEURO.0097-22.2023.f5-1 Extended Data Figure 5-1 Related to Figure 5. Analysis of motor and nonmotor behavior in neurorestoration paradigm. ***A***, Average velocity in the open field test. Two-way repeated measures ANOVA, Tukey’s multiple comparisons test. ***B***, Ratio of distance travelled in the center to the total travelled distance in the open field test. Two-way repeated measures ANOVA, Tukey’s multiple comparisons test. ***C***, Ratio of the time spent in the center to the total time in the open field test. Two-way repeated measures ANOVA, Tukey’s multiple comparisons test. ***D***, Rearing time in the open field test. Two-way repeated measures ANOVA, Tukey’s multiple comparisons test. ***E***, Average daily activity during the dark period in the home cage activity test over 7 d (26w). Two-way repeated measures ANOVA, Sidak’s multiple comparisons test. ***F***, Average daily activity during the light period in the home cage activity test over 7 d (26w). Two-way repeated measures ANOVA, Sidak’s multiple comparisons test. ***G***, Nest quality score in the home cage according to a five-point rating scale (26w). Unpaired *t* test. ***H***, Body weight measured from the baseline until eight weeks after LC injection. Two-way repeated measures ANOVA, Tukey’s multiple comparisons test. LC, lactacystin; WT, wild type; cHyper, conditional hypermorphic; SN, substantia nigra; STR, striatum; OF, open field; HCA, home-cage activity; BW, body weight. Download Figure 5-1, TIF file.

10.1523/ENEURO.0097-22.2023.f5-2 Extended Data Figure 5-2 Related to Figure 5. The effects of endogenous GDNF on IBA1 and GFAP immunoreactivity in neurorestoration paradigm. ***A***, The IBA1 stain density of the injected side relative to the noninjected side show a significant decrease in the nigral IBA1 immunoreactivity ratio of injected side/noninjected side of heterozygous animals as compared to wild-type animals (unpaired *t* test, *p < 0.05; N = mouse). ***B***, The GFAP stain density of the injected side relative to the noninjected side show no changes in nigral GFAP immunoreactivity ratio of injected side/noninjected side (unpaired *t* test, *p* > 0.05; N = mouse). ***C***, Representative images depicting IBA1-stained substantia nigra of both lactacystin-injected and noninjected sides of the same slice from both wild-type and heterozygous animals. ***D***, Representative images depicting GFAP-stained substantia nigra of both lactacystin-injected and noninjected sides of the same slice from both wild-type and heterozygous animals. Download Figure 5-2, TIF file.

### Analysis of endogenous GDNF deletion in the LC PD model

Next, we used *Gdnf* conditional knock-out (*Gdnf^cKO^*) mice crossed with Nestin-Cre mice (*Gdnf^cKO^* x Nestin-Cre) to study how CNS-specific deletion of endogenous GDNF affects sensitivity to LC-induced dopaminergic system damage. Because age is a predominant risk factor of PD and proteasome dysfunction (McNaught et al., 2002, 2003; Bentea et al., 2017; Pang et al., 2019), we tested the impact of loss of GDNF in 13- to 15-month-old (aged) mice. The mice were injected with LC into the right SN and monitored with behavioral tests for eight weeks (Experiment V; Fig. 7*A*). To be able to detect increased vulnerability to LC, and to prevent increased mortality in aged mice, we used a smaller dose of LC in this experiment. We found that the deletion of endogenous GDNF did not affect the severity of dopaminergic system lesion, as evident from a lack of difference between the number of amphetamine-induced ipsilateral rotations between the genotypes (Fig. 7*B*). In line with this observation, GDNF deletion in *Gdnf^cKO^* x Nestin-Cre mice had no effect on striatal dopamine levels after LC injection (Fig. 7*C*). Together, these results show that *Gdnf* deletion in the CNS does not increase the sensitivity of the dopaminergic system to LC-induced damage.

## Discussion

Rodent and nonhuman primate models of PD, which mostly include neurotoxins MPTP and 6-OHDA, have shown that ectopic GDNF alleviates motor symptoms, enhances dopamine levels in the brain, and protects dopamine neurons from degeneration (Hoffer et al., 1994; Kearns and Gash, 1995; Gash et al., 1996; Kearns et al., 1997; Oiwa et al., 2002; Kordower et al., 2006). Available data suggest that in the dopaminergic neurons of the midbrain RET receptor is essential to mediate GDNF neuroprotective and neurorestorative effects in MPTP-induced animal models (Kowsky et al., 2007; Drinkut et al., 2016; Conway et al., 2020; Conway and Kramer, 2022). Similarly, GDNF protected midbrain dopamine neurons from proteasome inhibitor lactacystin induced degeneration *in vitro* (Li et al., 2007) and *in vivo* (Du et al., 2008, 2013). Moreover, recent results from placebo-controlled clinical studies suggest that at least some patients benefit from ectopic GDNF delivery (Barker et al., 2020), providing rationale for further work in that direction.

Our previous analysis of *Gdnf^wt/Hyper^* mice constitutively overexpressing endogenous GDNF in a proteasome inhibitor LC induced PD model and on aging revealed that a 2-fold increase in endogenous GDNF enhances motor coordination and brain dopamine levels without side-effects (Kumar et al., 2015; Mätlik et al., 2018; Turconi et al., 2020). However, constitutively increased endogenous GDNF levels in these mice are also accompanied by developmental increase in the number of dopaminergic terminals in the striatum and dopamine cells in the SNpc (Kumar et al., 2015). Thus, it remained impossible to determine whether GDNF’s impact on dopaminergic system development contributed to the beneficial outcome and hence the therapeutic potential of adult-onset increase in endogenous GDNF remained unknown. Here, we asked whether adult-onset elevation of endogenous GDNF has therapeutic potential in PD treatment. To address this possibility and to bypass the developmental effects of endogenous GDNF overexpression, we used the conditional GDNF hypermorphic (*Gdnf^cHyper^*) mice (Mätlik et al., 2022).

We find that 2–4-fold adult onset increase in striatal endogenous GDNF levels does not protect or restore striatal dopamine levels or protect dopaminergic neurons in SNpc neither in the neuroprotection nor neurorestoration procedures in the LC-induced PD model. In addition, we find no gross differences between the genotypes in behavioral tests evaluating PD-related motor functions and nonmotor symptoms. Since age is the highest risk factor for PD (Pang et al., 2019) we next used LC in aged mice lacking GDNF specifically in the CNS (*Gdnf^cKO^* x Nestin-Cre). We find that CNS-specific deletion of endogenous GDNF does not make aged mice more vulnerable to LC-induced dopaminergic cell death.

The results of this study are in contrast with the body of literature on the effects of ectopic GDNF in various toxin models, predominantly MPTP and 6-OHDA, as well as some genetic models of PD (Barker et al., 2020; Chmielarz et al., 2020). Therefore, we would like to discuss possible explanations for the perceived discrepancy. In this study, we used the proteosome inhibitor LC to model PD. Administration of LC above the SNpc results in an accumulation of unfolded proteins and dopaminergic neuron degeneration (Bentea et al., 2015, 2017). We used LC instead of other commonly used toxins, such as MPTP and 6-OHDA for mainly two reasons. First, based on recent evidence, reduction in proteasome function is believed to cause or at least contribute to PD pathology, therefore LC targets a disease-relevant process (McNaught et al., 2002, 2003; Bentea et al., 2017). Second, it has been shown that both an increase and a decrease in endogenous GDNF levels in the striatum increase DAT activity, resulting in increased vulnerability of dopaminergic neurons to these toxins (Kumar et al., 2015; J.J. Kopra et al., 2017). Thus, using DAT-dependent toxins such as MPTP and 6-OHDA to model PD and simultaneously evaluate the effect of endogenous GDNF is not possible.

Finally, a potential explanation to differences observed using ectopic and endogenous GDNF overexpression could relate to the achieved levels of overexpression. In contrast to studies using ectopic GDNF delivery, which usually results in >10-fold or even >100-fold increase in striatal GDNF concentration (Tenenbaum and Humbert-Claude, 2017), endogenous GDNF upregulation in *Gdnf^cHyper^* mice is around 2–4-fold. A 3-fold overexpression of recombinant GDNF has been shown to be neuroprotective in a single study using 6-OHDA in monkeys (Eslamboli et al., 2005), and a 2-fold increase in GDNF protein expression from endogenous Gdnf mRNA results in similar rescue in 6-OHDA model mice (Espinoza et al., 2020). We show that a 2–4-fold increase in endogenous GDNF has little influence on LC-induced damage of the dopamine system. We observed a transient improvement in motor function on simultaneous injection of LC and GDNF upregulating AAV encoding for Cre recombinase, but no changes in dopamine or dopamine neuron numbers. These results suggest that on LC-mediated proteasome inhibition, 2–4-fold increase in endogenous GDNF may have transient neuroprotective effect but may not be sufficient to protect dopamine neurons from degeneration. We also assessed the effect of striatal ectopic GDNF delivery at levels exceeding endogenous GDNF expression ∼7–9-fold but similar to results from heterozygous and homozygous GDNF cHyper animals observed no effect in LC PD model. This suggests that in LC PD model GDNF levels are critical, as it has been shown that ectopic GDNF in high amounts (infused GDNF and AAV2-GDNF) is neuroprotective for SN dopaminergic neurons in lactacystin-treated mice (Du et al., 2008, 2013; d’Anglemont de Tassigny et al., 2015).

These results are in line with the lack of genetic connection between GDNF or its receptors and Parkinson’s disease and the observation that mice lacking GDNF and its receptors do not develop overt PD phenotypes (J. Kopra et al., 2015; Kumar et al., 2015). Furthermore, mice bearing the Met918Thr mutation in RET which yields constitutively active RET tyrosine kinase have robustly increased striatal dopamine and tyrosine hydroxylase levels but are not protected from 6-OHDA or MPTP induced PD (Mijatovic et al., 2007, 2008, 2011).

We conclude that modulation of endogenous GDNF at levels tested in this study does not impact dopamine neuron survival or function in a LC induced PD model. However, given that existing mouse models do not phenocopy the slow progression of PD, future studies pending on the generation of such models will be essential to definitively conclude on the therapeutic potential of endogenous GDNF in PD.

## Synthesis

Reviewing Editor: Eran Perlson, Tel Aviv University Decisions are customarily a result of the Reviewing Editor and the peer reviewers coming together and discussing their recommendations until a consensus is reached. When revisions are invited, a fact-based synthesis statement explaining their decision and outlining what is needed to prepare a revision will be listed below. The following reviewer(s) agreed to reveal their identity: NONE.

The manuscript entitled “Physiological GDNF plays no role in dopamine neuron protection and restoration in a proteasome inhibition model of Parkinson’s disease” reports that they fail to find any neuroprotective or neurorestorative effects of a GDNF hypermorphic allele that increases endogenous GDNF expression by 2-3 fold in an LC model of PD in adult mice. This result goes against previously published studies in mice, non-human primates, and patients. The main conclusion arrived by the authors is that either the dosage of GDNF afforded by their model was insufficient or the LC model differs from previous models, such as MPTP or 6OH-DA.

The manuscript should be carefully re-written and the title modified to put the carefully performed experiments into the proper scientific context without overstating the data. The correct statement in the title and abstract would be that upregulating endogenous GDNF levels in the striatum by 2-4 times the normal amount does not lead to protection and regeneration of midbrain dopaminergic neurons in a lactacystin mouse model. In addition, further experiments, clarifications, and crucial points of discussion are needed to interpret the data correctly in the context of the published literature.

Specific comments (not in order of importance):

1. The authors repeatedly state throughout the manuscript that previous clinical trials of PD patients with GDNF have been unsuccessful. Such a sweeping statement is not warranted as there is indeed evidence of beneficial effects in several patients treated with GDNF. The authors are recommended to adopt a more balanced view when making such assertions.

2. The authors use injection of proteasome inhibitor LC to model PD. The conclusions of their study can go only as far as the suitability of this PD model, which, like with several other models of human PD, has proven to be somewhat limited.

3. Reference in lines 54/55: In addition to this reference, Mart Saarma has reviewed this topic specifically and should be cited here.

4. The approach using CRE-expressing viral vectors to trigger conditional allele recombination is contingent on successful and accurate viral targeting, both location and amount, and induction of recombination. Because the results reported by the authors are negative, namely lack of effects, the viral approach should be complemented with endogenous CRE expression from, for example, the DAT-CreERT2 locus.

5. The authors may wish to explain the use of the AAV5 virus serotype instead of others most commonly used for in vivo studies, namely, AAV2 and AAV9.

6. Since one of the main issues discussed by the authors is that the failure of previous clinical trials may have been due to mislocalization of the exogenous GDNF, analysis of bulk protein expression should be complemented with immunohistochemistry to verify the cellular localization of GDNF overexpression. In this regard, analysis of the localization of the virus after injection would also be helpful to validate the accuracy of the injections.

7. The levels of GDNF mRNA and protein should also be assessed subsequently to LC injection in experiments II, III, and IV to determine whether there were any detrimental effects in GDNF expression induced by the drug. This would be an alternative explanation to the “lack of effect” of GDNF overexpression as claimed by the authors.

8. In the behavioral studies, it would be helpful to show the performance of mice (WT and mutants) treated with a vehicle, a common control in this type of experiments. Without a vehicle control, it is impossible to assess the extent of the lesion introduced by LC injection in each genotype, limiting the interpretation of the results.

9. How do the authors explain the temporary improvement in motor function observed upon GDNF overexpression if neither DA levels nor DA cell bodies were preserved in those mice? This result suggests that GDNF does have a neurotrophic effect on these neurons but that the levels achieved in this model are insufficient to protect the neurons. It is known from the field that different neurotrophic effects can be elicited at different dosage thresholds. Maybe GDNF would effectively rescue DA levels and neurons if it were overexpressed at higher levels. Homozygous hypermorphic mice should be tested in all paradigms of LC injection (II and III, in addition to IV).

10. GDNF mRNA and protein levels should be measured in GdnfcKO x Nestin-Cre mice. How substantial was the reduction? How much residual GDNF remains in these mice? These data are essential for proper interpretation of the results.

11. The authors discuss several possible advantages of the LC model of PD versus other models, but they have not verified here that, in their hands, LC injection did indeed impair proteasome function or led to increased accumulation of unfolded proteins or did not impair DAT activity. Whether this happened in their studies remains speculative.

12. The size of all graphs in the figures are too small and needs to be increased.

13. Also, midbrain dopaminergic neurons of the VTA region should be counted, and the effect of lactacystin and GDNF compared to SN dopaminergic neurons.

14. The significance should be shown as stars in figure 1F.

15. Higher amounts of GDNF should be provided to lactacystin-treated mice, for example, by stereotactic injection of GDNF protein or GDNF encoding viruses, to challenge the data from Du et al. 2008 and Du et al. 2013 and to support the idea that the low amounts of GDNF prevented to observe a neuroprotective and regenerative effect.

16. The effect of GDNF on lactacystin-induced alpha-synuclein accumulation, inflammation, and gliosis should be investigated (Savolainen et al., 2017).

Finally, the authors need to include and discuss at least the following literature:

Li et al., 2007, described that rat ventral mesencephalic cell cultures treated with lactacystin were protected by GDNF from degeneration by supressing endoplasmatic reticulum stress and caspase-3 activation.

Du et al., 2008, show that infused GDNF is neuroprotective in vivo for SN dopaminergic neurons in lactacystin-treated mice.

In Du et al., 2013, they repeated their experiments with AAV2 expressed GDNF and could convincingly show that GDNF is neuroprotective and leads to neuroregeneration of SN dopaminergic neurons in lactacystin-treated mice.

Tassigny et al., 2015, mentioned these data also in their review.

Kowsky et al., 2007, have also shown that endogenous GDNF/Ret signalling can mediate the regeneration of SN dopaminergic neurons in MPTP treated mice.

More recently, Drinkut et al., 2016, has shown that AAV2 expressed GDNF protects from MPTP-induced midbrain dopaminergic neurons toxicity in mice and also promotes regeneration.

These data are nicely reviewed by Conway et al., 2020 and Conway et al., 2022.

The data on GDNF receptor Ret knockout mice (Kowsky et al., 2007; Drinkut et al., 2016) and MEN2B mice (Mijatovic et al., 2008; Mijatovic et al., 2011) are especially interesting to discuss in the context of the presented GDNF knockout data.
