# Supplementary figures and images for: Increased Physiological GDNF Levels Have No Effect on Dopamine Neuron Protection and Restoration in a Proteasome Inhibition Mouse Model of Parkinson’s Disease
Source: eNeuro. 2023 Feb 7;10(2):ENEURO.0097-22.2023. doi: 10.1523/ENEURO.0097-22.2023 (PMC9910577; doi:10.1523/ENEURO.0097-22.2023)

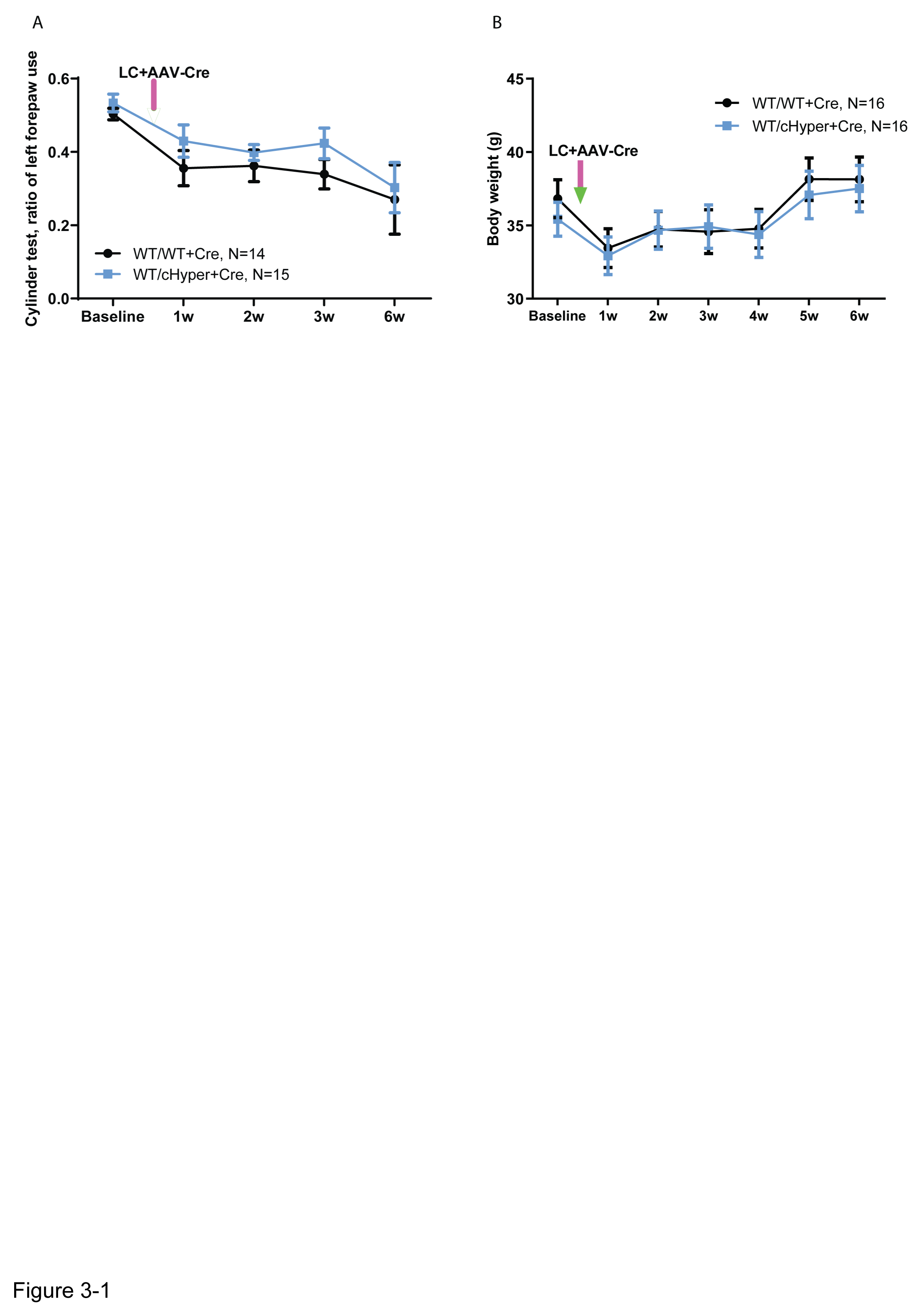

Supplement: Extended Data Figure 3-1 — Related to Figure 3. Elevation of GDNF expression levels after simultaneous AAV-Cre and LC injection and the effects on cylinder test and body weight. A, Ratio of left forepaw use in the cylinder test one, two, three, and six weeks after LC and AAV-Cre simultaneous injection. Two-way repeated measures ANOVA, Sidak’s multiple comparisons test. B, Body weight from the baseline until the end of the experiment. Two-way repeated measures ANOVA, Sidak’s multiple comparisons test. LC, lactacystin; WT, wild type; cHyper, conditional hypermorphic; STR, striatum; Cyl, cylinder test; BW, body weight. *p < 0.05, **p < 0.01, ***p < 0.001. Download Figure 3-1, TIF file. [file enu-eN-NRS-0097-22-s02.tif]

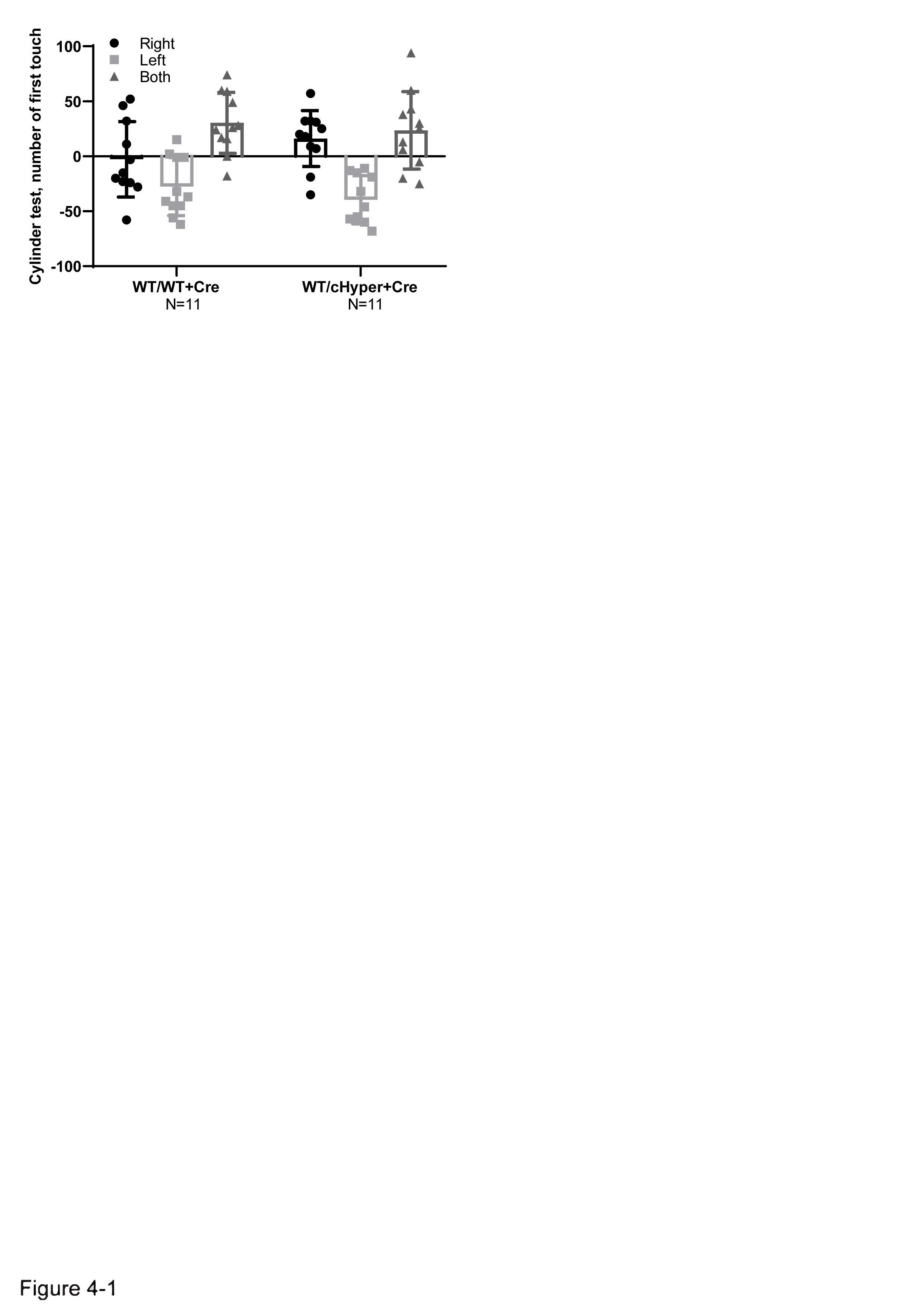

Supplement: Extended Data Figure 4-1 — Related to Figure 4. Analysis of motor behavior in neuroprotection paradigm. Number of first touches in the cylinder test made with either right, left or both forepaws (3 weeks after LC). WT, wild type; cHyper, conditional hypermorphic; STR, striatum; Cyl, cylinder test. Download Figure 4-1, TIF file. [file enu-eN-NRS-0097-22-s03.tif]

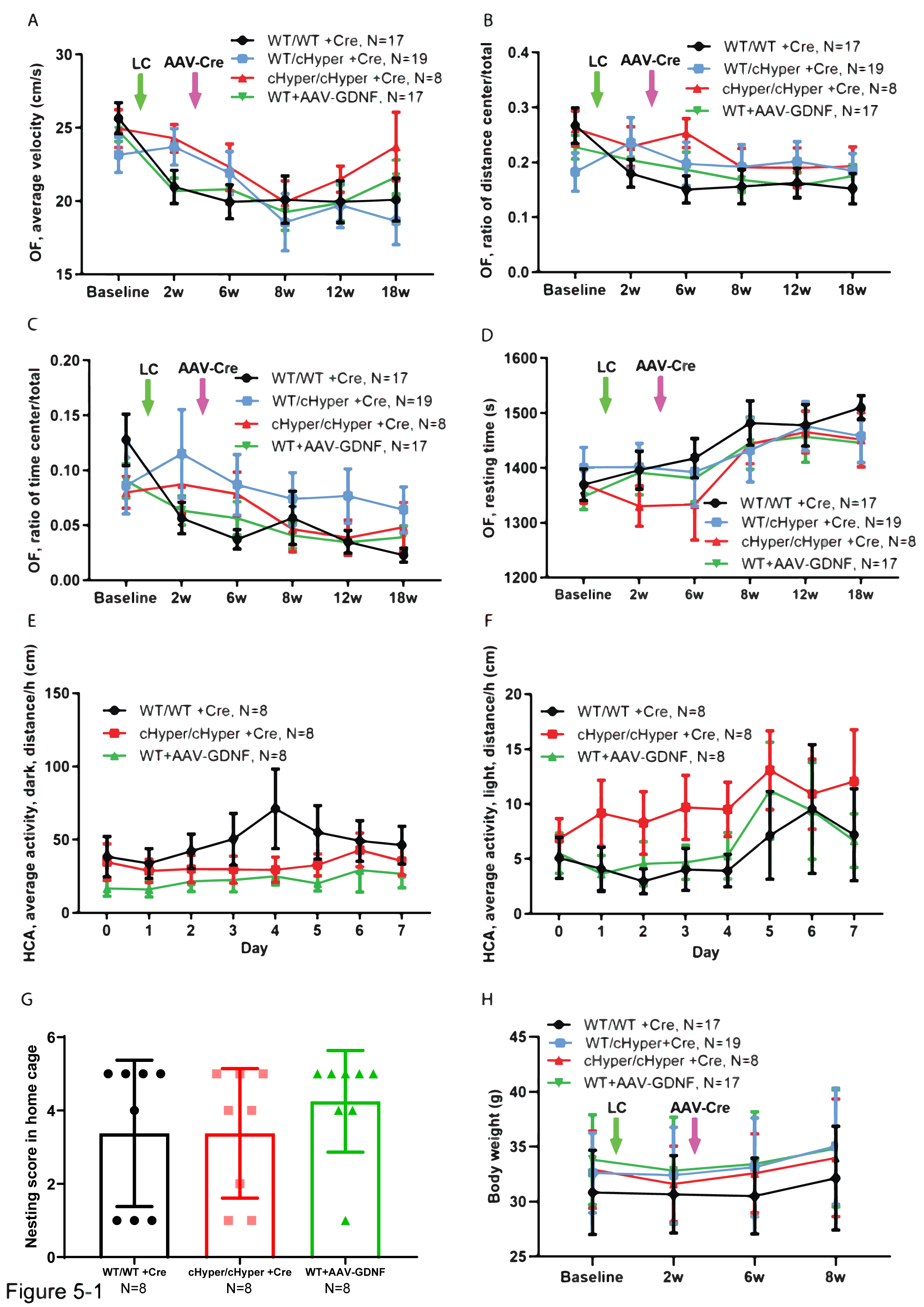

Supplement: Extended Data Figure 5-1 — Related to Figure 5. Analysis of motor and nonmotor behavior in neurorestoration paradigm. A, Average velocity in the open field test. Two-way repeated measures ANOVA, Tukey’s multiple comparisons test. B, Ratio of distance travelled in the center to the total travelled distance in the open field test. Two-way repeated measures ANOVA, Tukey’s multiple comparisons test. C, Ratio of the time spent in the center to the total time in the open field test. Two-way repeated measures ANOVA, Tukey’s multiple comparisons test. D, Rearing time in the open field test. Two-way repeated measures ANOVA, Tukey’s multiple comparisons test. E, Average daily activity during the dark period in the home cage activity test over 7 d (26w). Two-way repeated measures ANOVA, Sidak’s multiple comparisons test. F, Average daily activity during the light period in the home cage activity test over 7 d (26w). Two-way repeated measures ANOVA, Sidak’s multiple comparisons test. G, Nest quality score in the home cage according to a five-point rating scale (26w). Unpaired t test. H, Body weight measured from the baseline until eight weeks after LC injection. Two-way repeated measures ANOVA, Tukey’s multiple comparisons test. LC, lactacystin; WT, wild type; cHyper, conditional hypermorphic; SN, substantia nigra; STR, striatum; OF, open field; HCA, home-cage activity; BW, body weight. Download Figure 5-1, TIF file. [file enu-eN-NRS-0097-22-s04.tif]

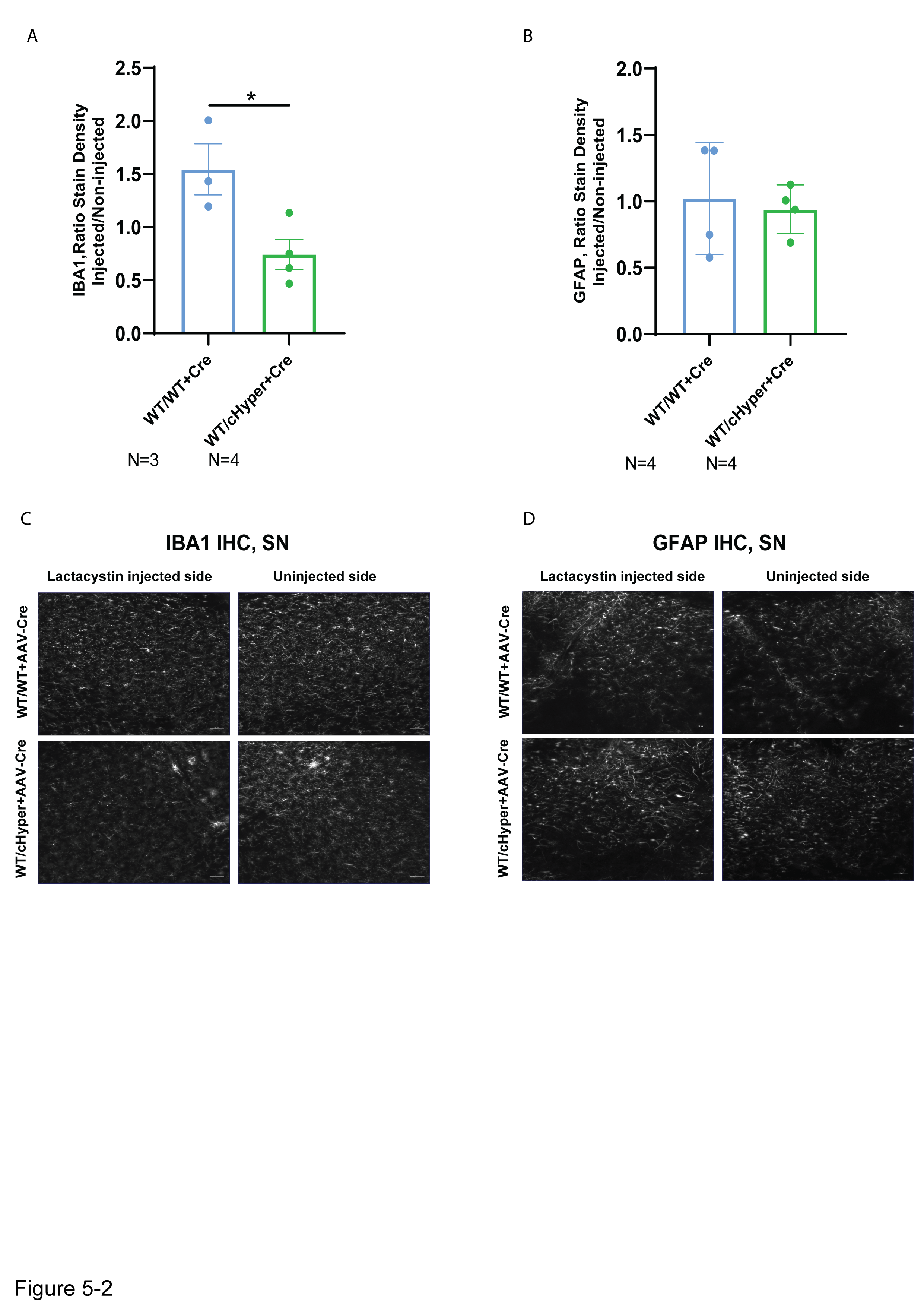

Supplement: Extended Data Figure 5-2 — Related to Figure 5. The effects of endogenous GDNF on IBA1 and GFAP immunoreactivity in neurorestoration paradigm. A, The IBA1 stain density of the injected side relative to the noninjected side show a significant decrease in the nigral IBA1 immunoreactivity ratio of injected side/noninjected side of heterozygous animals as compared to wild-type animals (unpaired t test, *p < 0.05; N = mouse). B, The GFAP stain density of the injected side relative to the noninjected side show no changes in nigral GFAP immunoreactivity ratio of injected side/noninjected side (unpaired t test, p > 0.05; N = mouse). C, Representative images depicting IBA1-stained substantia nigra of both lactacystin-injected and noninjected sides of the same slice from both wild-type and heterozygous animals. D, Representative images depicting GFAP-stained substantia nigra of both lactacystin-injected and noninjected sides of the same slice from both wild-type and heterozygous animals. Download Figure 5-2, TIF file. [file enu-eN-NRS-0097-22-s05.tif]
